# Impact of a Single Bout of Aerobic Exercise on Regional Brain Perfusion and Activation Responses in Healthy Young Adults

**DOI:** 10.1371/journal.pone.0085163

**Published:** 2014-01-08

**Authors:** Bradley J. MacIntosh, David E. Crane, Michael D. Sage, A. Saeed Rajab, Manus J. Donahue, William E. McIlroy, Laura E. Middleton

**Affiliations:** 1 Department of Medical Biophysics, University of Toronto, Toronto, Ontario, Canada; 2 Canadian Partnership for Stroke Recovery, Sunnybrook Research Institute, Toronto, Ontario, Canada; 3 Toronto Rehabilitation Institute, University of Toronto, Toronto, Ontario, Canada; 4 Graduate Department of Rehabilitation Science, University of Toronto, Toronto, Ontario, Canada; 5 Department of Kinesiology, University of Waterloo, Waterloo, Ontario, Canada; 6 Department of Radiology, Vanderbilt University, Nashville, Tennessee, United States of America; University of Texas Southwestern Medical Center, United States of America

## Abstract

**Purpose:**

Despite the generally accepted view that aerobic exercise can have positive effects on brain health, few studies have measured brain responses to exercise over a short time span. The purpose of this study was to examine the impact within one hour of a single bout of exercise on brain perfusion and neuronal activation.

**Methods:**

Healthy adults (n = 16; age range: 20–35 yrs) were scanned using Magnetic Resonance Imaging (MRI) before and after 20 minutes of exercise at 70% of their age-predicted maximal heart rate. Pseudo-continuous arterial spin labeling (pcASL) was used to measure absolute cerebral blood flow (CBF) prior to exercise (pre) and at 10 min (post-10) and 40 min (post-40) post-exercise. Blood oxygenation level dependent (BOLD) functional MRI (fMRI) was performed pre and post-exercise to characterize activation differences related to a go/no-go reaction time task.

**Results:**

Compared to pre-exercise levels, grey matter CBF was 11% (±9%) lower at post-10 (P<0.0004) and not different at post-40 (P = 0.12), while global WM CBF was increased at both time points post-exercise (P<0.0006). Regionally, the hippocampus and insula showed a decrease in perfusion in ROI-analysis at post-10 (P<0.005, FDR corrected), whereas voxel-wise analysis identified elevated perfusion in the left medial postcentral gyrus at post-40 compared to pre (p_corrected_ = 0.05). BOLD activations were consistent between sessions, however, the left parietal operculum showed reduced BOLD activation after exercise.

**Conclusion:**

This study provides preliminary evidence of regionalized brain effects associated with a single bout of aerobic exercise. The observed acute cerebrovascular responses may provide some insight into the brain’s ability to change in relation to chronic interventions.

## Introduction

Aerobic exercise has positive effects on the brain, as exemplified by meta-analytic data showing that exercise improves cognitive function [1] and reduces stroke risk [2]. An aerobic exercise training program improves endothelial function and vascular resistance and reduces vascular risk factors [3]. The potential benefits of exercise to brain health are demonstrated by an increase in brain volume in the anterior cingulate, supplementary motor area, right inferior frontal gyrus and the left superior temporal gyrus [4] as well as the hippocampus [5]. Vascular changes are an important substrate of neuroplastic change and believed to be one of the core components of exercise-associated changes [6]–[9].

The cerebrovascular effect of aerobic exercise training has been quantified in animal models and, at a preliminary level, in humans. Treadmill training paradigms have been used in rats [10] and primates [11] to show increases in regional CBF and vascular density in cortex, respectively. In humans, cerebrovascular reactivity is increased among stroke survivors after 6-months of aerobic training [12] and among older women with better aerobic fitness [13]. Another study reported an increase in cerebral blood volume in the dentate gyrus with aerobic exercise training [14]. By contrast, the limited research regarding the influence of a single bout of exercise (i.e. acute influences) has focused mainly on cognition [15] and neurophysiological effects [16], [17]. Of the few studies that have evaluated the cerebrovascular effects of acute exercise, the emphasis has been on effects during exercise with less attention to post-exercise effects [18], [19].

The purpose of the present study was to determine whether changes in cerebrovascular control persist after the end of a single bout of exercise. The primary objective of this study was to use whole brain pseudo-continuous arterial spin labeling (pcASL) to determine whether exercise can induce regional and time-dependent perfusion changes. Our secondary objective was to evaluate whether exercise will elicit any changes in task-related functional activity using blood oxygenation level dependent (BOLD) contrast. We hypothesized that these cerebrovascular measures would reveal acute effects of exercise on the brain in a cohort of healthy young adults. Understanding of the effects of a single-bout of aerobic exercise is an important area of research because these findings may relate to neuroplastic changes that are reported in exercise interventions.

## Methods

### 2.1 Participants and experimental design

Sixteen healthy adults between the age of 20 and 35 years participated in this study (see Table 1 for details). A brief questionnaire was used to gauge physical activity levels and consisted of the following: 1) Do you do physical activity in a week? 2) How many times per week? 3) How many minutes per session? 4) What type exercise do you do? Participants performed a single bout of aerobic exercise, along with same day pre and post-exercise MRI scans. The Sunnybrook Health Sciences Centre Ethics Board approved this study and all participants provided signed informed consent.

**Table 1:** Characteristics of the study sample.

Characteristics	Mean (SD) or % (n).
N	16
Age, yrs	26.7 (4.1)
Sex, % Female	62.5% (10)
Education, yrs	17.6 (2.6)
Handedness, % right	81.3% (13)
Height, m	171.0 (10.5)
Exercise sessions per week, #	3 (1.7)
Duration of exercise session, minutes	45 (20.7)
Weight, kg	67.9 (13.9; range: 50 – 100)
Resting heart rate, BPM	72.4 (22.2)
Exercise heart rate, BPM	135.8 (3.7)
Average predicted HR, %	70.3 (5.3)
Work rate, Watts	86 (33; range: 49 – 176)
Last RPE	4.5 (1.8)

### 2.2 Exercise

Participants performed 25 minutes of work on a stationary, recumbent bicycle ergometer. This included 3 minutes of self-paced warm up, 20 minutes of exercise at 70% of the age-predicted maximal heart rate (HR), and a 2-minute cool down. Age-predicted maximal HR was defined as 220 beats-per-minute (BPM) minus age in years [20]. Participants were informed of their target heart rate prior to starting the exercise session and were given feedback if they strayed more than 5 bpm from their target heart rate. HR was monitored continuously during exercise and recorded every minute. During exercise, Borg ratings of perceived exertion (RPE) were provided every 10 minutes [21], and systolic and diastolic blood pressure (SBP/DBP, mmHg) were recorded before and after the exercise session.

### 2.3 Magnetic resonance imaging

MRI was performed on a 3 Tesla Philips Achieva MRI system using body coil transmission and an 8 channel head receive coil. The MRI protocol consisted of: 1) T1-weighted (T1w) high resolution fast-field echo imaging (TR/TE/TI = 9.5/2.3/1400, spatial resolution 0.94×1.2×1.2mm, 256×164×140 matrix, scan duration 8:38 mins); 2) time-of-flight angiography (TOF; TR/TE = 25/3.5, spatial resolution 0.4×0.7×1.4 mm, 496×283×88 matrix, scan duration 3:30 mins); 3) gradient echo images with blood oxygenation level dependent (BOLD) T2*-weighted contrast echo planar imaging (EPI) (TR/TE =  1500/30 ms; 230 volumes, task details are below); 4) perfusion imaging with pseudo-continuous arterial spin labeling (pcASL) with single shot EPI (TR/TE =  4000/9.7 ms, 64×64×18 matrix, spatial resolution 3×3×5 mm, 1650 ms labeling duration (LD), post-label delay  = 1600 ms, 35 control and tag pairs, scan duration of 4:48 mins) [22]. TOF images were used to prescribe the labeling region that was perpendicular to the internal carotid arteries, approximately 80 mm below the lowest pcASL slice and located typically between C1 and C2 cervical vertebrae. The pre-exercise MRI scan order was: survey, TOF, pcASL, BOLD-fMRI, pcASL reference. The post-exercise scan order was: survey, post-10 pcASL, BOLD-fMRI, T1, pcASL reference, post-40 pcASL.

### 2.4 Arterial Spin Labeling: acquisition & analysis

Perfusion data were collected: once at baseline (pre), another 10 minutes (post-10) and a final one at 40 minutes (post-40) post-exercise. Additional short scan duration ASL images were collected with a long TR (10 s) and at 5 separate TEs (i.e. 9.6, 20, 30, 40, 80 ms) to estimate the initial magnetization, T_2_^*^ and extract the RF receiver coil sensitivity profile across the imaging volume.

ASL data were analyzed using tools available in FMRIB Software Library (FSL) and in-house software scripts. Co-registration of the ASL time series data was done using MCFLIRT [23]. Difference images were smoothed spatially using a 5 mm full-width half maximum kernel. As described previously [22], absolute CBF (units: mL/100 g/min) quantification was calculated based on the following equation for a single tissue compartment model: 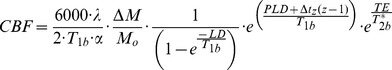
where 60 converts seconds to minutes, 100 is a conversion factor to 100 g of tissue, ▵M is the ASL difference signal, α = 0.85 is the labeling efficiency, λ = 0.9 is the blood brain barrier partition coefficient, M_0_ is the equilibrium magnetization signal of blood, T_1,b_ = 1.68s is the T_1_ of arterial blood, T_2*,t_ = 0.050s is the effective relaxation time of grey matter, TE = 0.0097s is the echo time, PLD  = 1.6s is the post-labeling delay, and Δt_z_ = 0.036s and z goes from slice 1 to 18 . A mono-exponential model was used to fit T_2*_ and M_0_ as parameters to the multi-TE saturation recovery ASL reference data, performed using Matlab (Matlab, Mathworks, Natick, MA). M_0_ was estimated from voxels in the CSF that were selected manually and converted to M_0_ using a saturation recovery equation and a difference in proton density between blood and CSF of 0.76. Grey matter (GM), white matter (WM) and CSF tissue types were segmented using the FMRIB Automated Segmentation Tool (FAST) from the T1-weighted image and 90% probability threshold masks were transformed to ASL space.

### 2.5 Sustained attention to response task & fMRI

Task-related BOLD fMRI involved a go/no-go reaction time block-design implementation of a modified sustained attention to response task (SART) [24] with alternating blocks of 30 s of task and 20 s rest for 5:45 minutes. The task was designed in E-prime (Psychology Software Tools, Inc.) and stimuli were projected onto a screen at the foot of the scanner that participants viewed through a mirror mounted on the head coil. Digits 1 to 9 were presented in pseudorandom order, varying font type (Arial, Courier New, Georgia, Kartika, Veranda) and font sizes (5 sizes) over the six blocks. Stimuli were presented for 250 ms (black font, white background) and followed by a 1.15 s fixation cross. Participants were instructed to respond as quickly and accurately as possible to “go” trials with a right index finger button press (Nordic Neuro Labs ResponseGrip, Bergen, Norway). “Go” trials were all digits except for digit 3, which was the “no-go” trial. Rest conditions consisted of a black ring with a diagonal cross through the middle. Participants performed a practice test just prior to the first scanning session.

Behavioural data were assessed in terms of accuracy and reaction time (RT). Two accuracy measures were used: 1) Errors of commission (key presses during a “no-go” trials); and 2) errors of omission (lack of key press during a “go” trial). RT was assessed as: 1) the RT for correct responses (ms); and 2) between trial RT-variability, calculated as standard deviation of the RT for correct responses divided by the mean RT for correct responses [25].

BOLD-fMRI data were analyzed as a block-design using the FMRI Expert Analysis Tool (FEAT) in FSL with the following processing steps: motion correction, brain extraction, spatial smoothing (8 mm), register fMRI to T1 images with 7 degrees of freedom (DOF) followed by a 12 DOF co-registration of T1 to MNI standard space.

### 2.6 Statistical analysis

For the CBF data, a one-way analysis of variance test was used to establish an overall session effect and paired t-tests were used to compare the 3 time points (i.e. pre vs post-10, pre vs post-40 time points). These analyses were performed on the absolute perfusion images using: 1) region of interest (ROI) and 2) voxel-wise approaches. For the ROI approach, whole brain, global GM and global WM CBF values were used to assess global trends and ROIs in the caudate, hippocampus, insula, middle frontal gyrus, postcentral gyri, precentral gyri regions were chosen to detect regional effects and based on the exercise intervention human neuroimaging literature [4], [10], [14], [26]. A false discovery rate (FDR) of q = 0.05 was calculated for ANOVA and t-tests separately and used to correct for multiple ROI comparisons. For voxel-wise analyses, a permutation based algorithm called randomise was chosen for its ability to accommodate parametric or non-parametric data [27], as described in a previous ASL study [28]. Voxel level p-values were corrected for multiple comparisons at p = 0.05 (p_corr_) using 5000 permutations and threshold free cluster enhancement (height and extent settings of 4 and 0.4, respectively).

Paired t-tests were also performed to test for a session effect on the behaviour and fMRI activation data, Spearman’s correlations (r) were used to correlate RT and error rate and a mixed regression was used to assess the influence of session and error on RT. FMRI group analyses were performed using a mixed-effects ordinary least squares model using clusters determined by Z>1.96 and a corrected cluster significance threshold of P = 0.05. In the fMRI analysis, CBF images at pre and post-10 time points were included as a voxel-wise covariate.

## Results

### 3.1 Participant demographics

Participants (10 women, 6 men) had a mean age of 26.7 (SD 4.1) years. All but one participant reported regular physical activity throughout the course of the week, through leisure activities (N = 14) or physical labour (N = 1). Types of exercise included jogging, running, cycling, weights, soccer and others, on average 3.0 (SD 1.7) times per week for 45 (SD 21) minutes per session. Additional characteristics are presented in Table 1.

### 3.2 Global exercise effects

**Figure 1:**
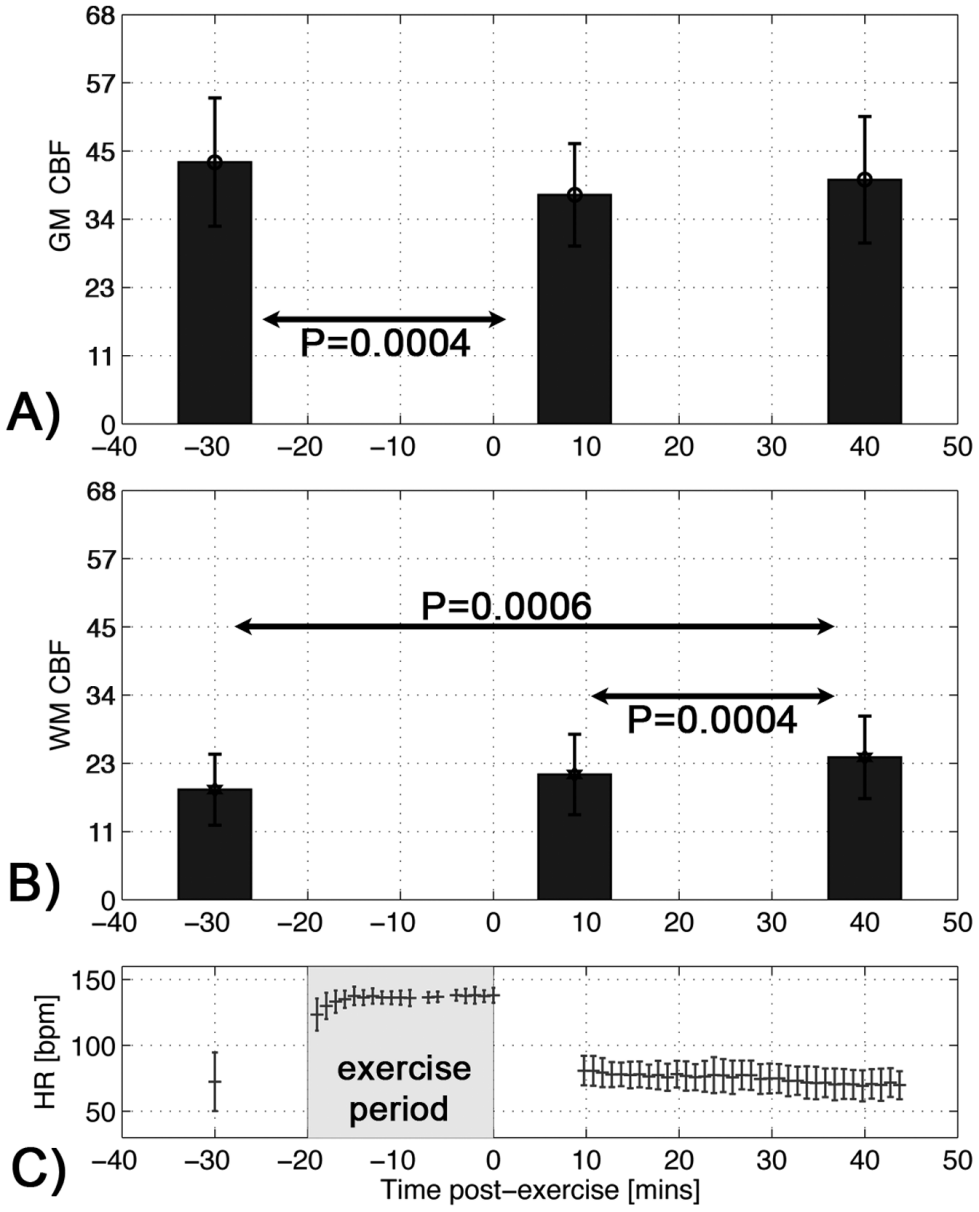
Cerebral blood flow measures before and after exercise. A) Global grey matter and B) white matter CBF values [mL/100 g/min] are plotted versus time. Post-10 GM-CBF was significantly reduced compared to baseline. Post-40 had higher WM CBF compared to post-10 and pre-exercise levels. C) The average HR data (with error bars as SD) are shown at pre, during and post-exercise. The rectangular box denotes the exercise period. Mean values are plotted with standard deviation as error bars.

The mean HR during exercise was 135.8 (SD 3.7) bpm, which corresponded to an average of 70.3% (SD 5.3%) of age-predicted maximal HR across subjects. Average HR across time is shown in Figure 1C. Systolic blood pressure (SBP) at pre, post-10 and post-40 exercise was 110.3 (SD 14.5), 117.6 (SD 12.7), 110.1 (SD 11.1) mmHg, respectively. Diastolic blood pressure (DBP) at pre, post-10 and post-40 exercise was 72.8 (SD 9.7), 72.0 (SD 8.6), 68.2 (SD 7.0) mmHg, respectively. From these data, SBP post-10 > pre was significantly different (P = 0.006). The average RPE at the end of the exercise bout was 4.5 (SD 1.8), which is within a range described by “somewhat hard” to “hard”. Additional physiological data are found in Table 1.

### 3.3 The effect of session on perfusion

Mean (SD) GM CBF was 43 (9.4) pre-exercise, 39 (7.5) at post-10 and 41 (9.3) mL/100 g/min at post-40 (Figure 1A). GM CBF was not significant for overall sessions (F[2], [45] = 1.20, P = 0.31) but showed a significant contrast of pre > post-10 (p = 0.0004). Contrasts for post-40 vs pre-exercise and post-40 vs post-10 were not significant (P = 0.13, P = 0.085, respectively). Mean WM CBF was 18 (5.2) pre-exercise, 20 (5.9) at post-10 and 24 (6.0) mL/100 g/min at post-40 (Figure 1B). WM CBF was not significant for session (F[2], [45] = 2.74,P = 0.08) nor post-10 > pre (P = 0.08) but was significantly elevated at post-40 vs pre-exercise (P = 0.0006) as well as post-40 vs post-10 (P = 0.00004). The whole brain CBF data were not significant on either ANOVA (P>0.60) or t-test levels (P>0.30).

**Figure 2:**
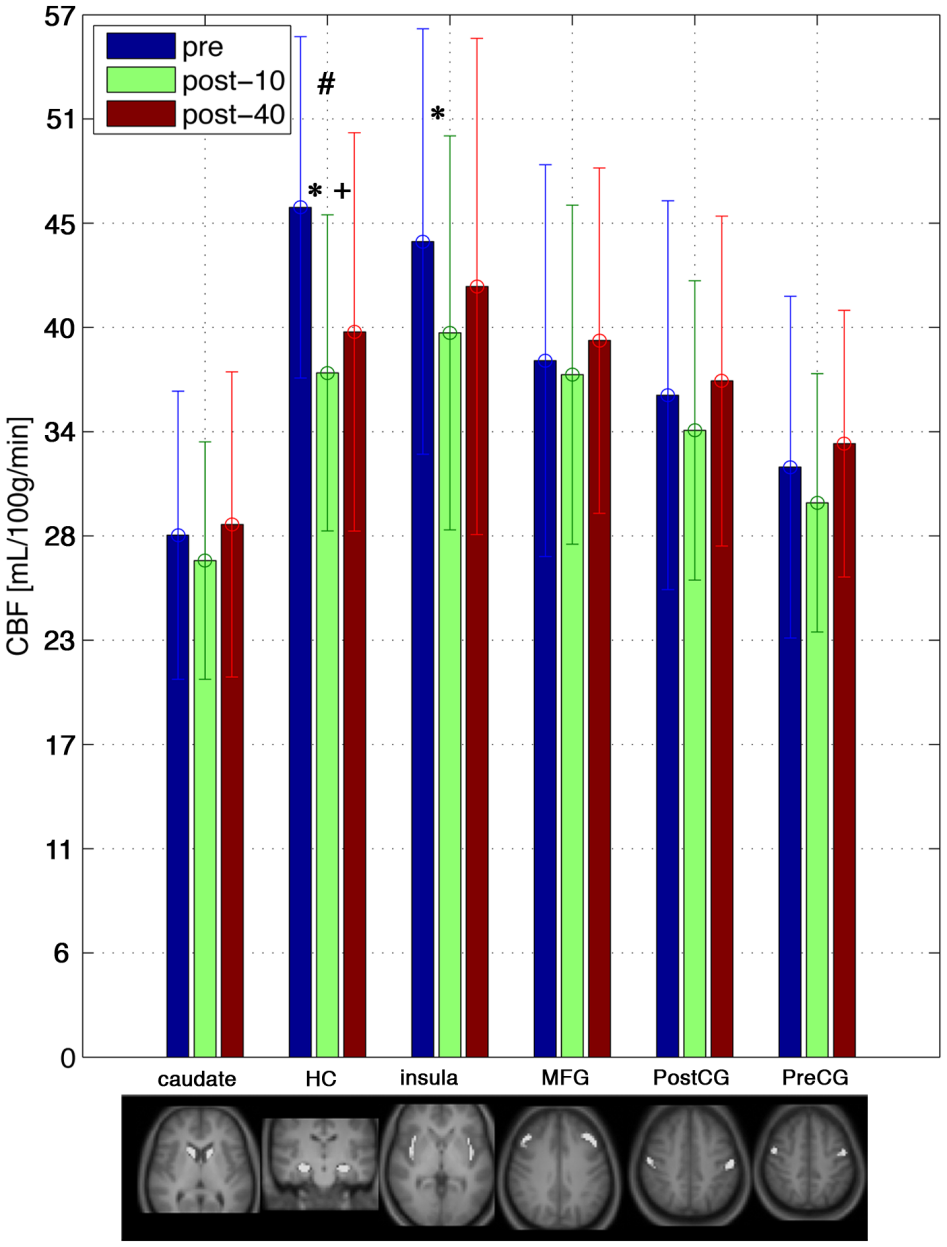
ROI analyses revealed a significant CBF session effect in the hippocampus, denoted by # for the uncorrected one-way ANOVA. In the paired differences, the hippocampus and insula regions were different for the different time points. * denotes pre > post-10 and + denotes pre < post-40 significant t-tests after FDR correction. The masks for the ROIs are shown in the bottom panels (HC = hippocampus, MFG = middle frontal gyrus, CG = central gyrus).

**Figure 3:**
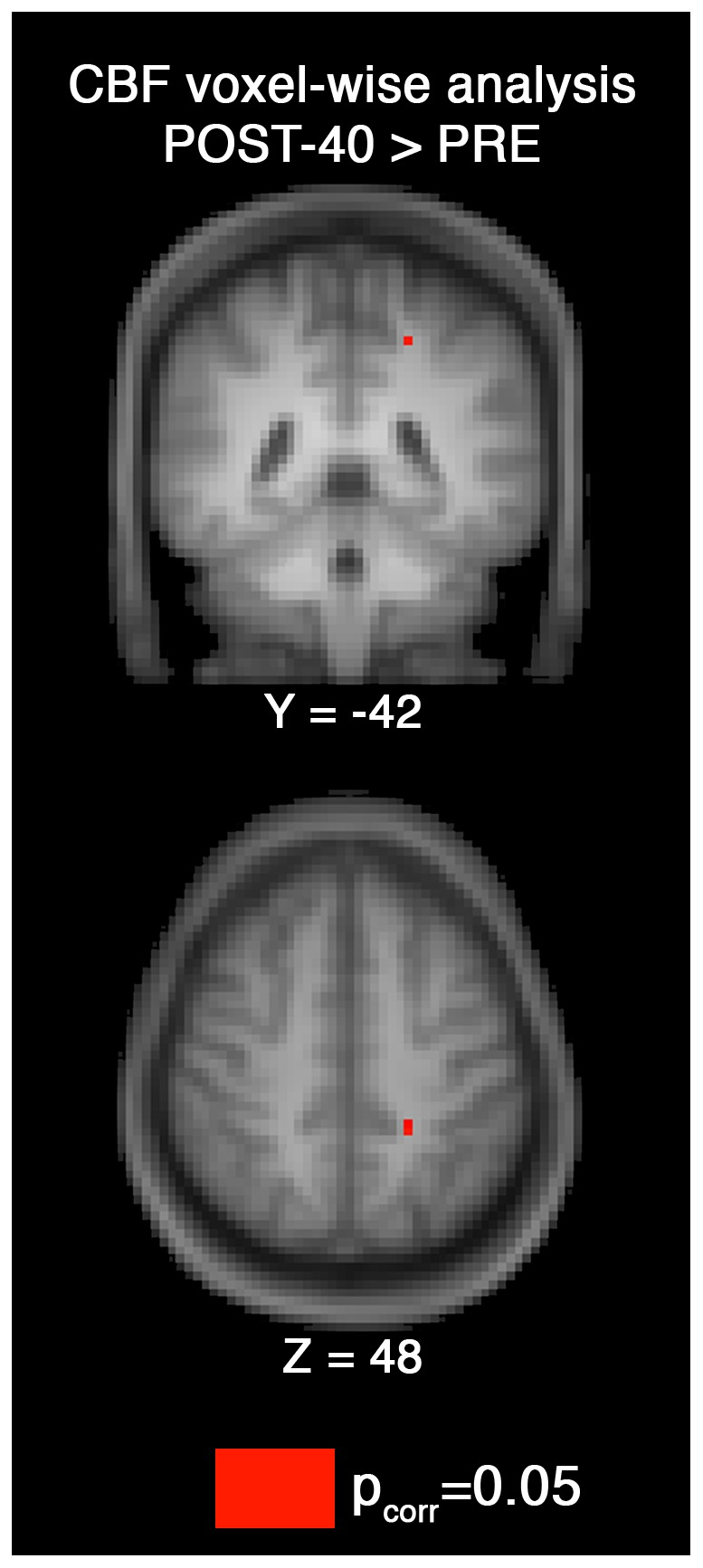
The voxel-wise analysis identified a single brain region that had increase CBF 40 minutes after exercise compare to pre-exercise levels (corrected for multiple comparisons at P_corrected_ = 0.05). The MNI coordinate was X = –21 mm, Y = –42 mm, Z = 48 mm, along the left medial bank of the post central gyrus, a somatosensory region for the lower limb.

The hippocampus ROI showed a session effect trend (F[2], [45]  = 3.8,P = 0.03) that did not survive an FDR correction. As seen in Figure 2, the paired CBF t-tests were significant, and survived an FDR correction, in the hippocampal ROI for pre > post-10 (t = 4.32, P =  0.0006) and pre > post-40 (t = 2.88, P = 0.01) and the insula ROI for pre > post-10 (t = 3.33, DOF = 15, P =  0.005). No voxel clusters were identified in the voxel-wise analysis for the session effect (i.e. P_corrected_>0.05), however, the paired contrasts showed post-40 > pre in a left medial post central region (in proximity to the sensorimotor cortex), located at X = –21 mm, Y = –39 mm, Z = 48 mm in MNI standard space (P_corrected_ = 0.05; Figure 3).

### 3.4 The effect of session on task-based BOLD fMRI

**Figure 4:**
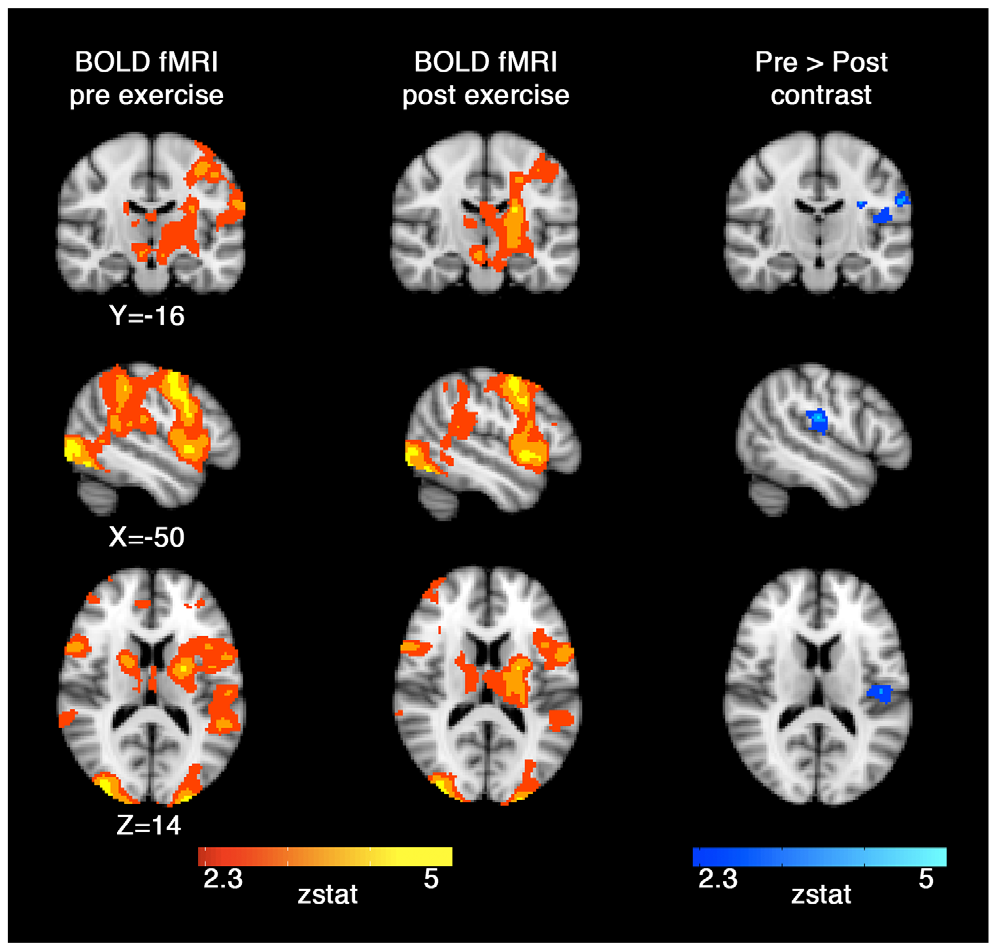
BOLD activation for the group during the pre-exercise fMRI (left). BOLD activation for the group after exercise (middle). The main task effect z-stat maps are shown as the red to yellow colour scale. The left parietal operculum was identified as significantly different on the paired session effect as seen on the blue colour scale z-stat map (right). Images are shown in radiological convention.

There was no effect of session on accuracy or RT (P>0.36; see Table 2). Reaction time and the number of errors of commission were highly correlated between participants (Spearman’s r = –0.84, P<0.01), a trend that was independent of session. Specifically, every additional error was associated with an 8.1 ms decrease in reaction time (P<0.001). Individual sessions produced significant fMRI activation in multiple bilateral brain regions including the visual and sensorimotor cortices, thalamus, putamen, anterior cingulate and middle frontal gyri. The pre and post-exercise sessions activation z-stat maps are shown in Figure 4 (red-yellow regions; P_corrected_ = 0.05). A paired comparison revealed that the left parietal operculum had significantly greater BOLD activation before exercise compared to post-exercise (blue regions; pre > post, P_corrected_ = 0.05, Figure 4).

**Table 2:** Behavioural data from the sustained attention to response task (SART).

Measure	Mean (SD) pre-exercise	Mean (SD) post-exercise	paired p-value
Errors of Commission	38.0 (17.0)	37.7 (20.0)	0.96
Errors of Omission	0.1 (0.3)	0.1 (0.2)	0.75
RT Correct Responses	325.8 (35.6)	326.4 (45.6)	0.97
Intra-individual SD	61.8 (10.5)	62.1 (12.0)	0.94

## Discussion

This study revealed that a single bout of aerobic exercise is associated with cerebrovascular changes in resting perfusion (ASL) and task-related activation (BOLD fMRI) measures in healthy adults. Global GM and WM data showed significant time-dependent changes, with GM CBF decreasing at 10-minutes post-exercise and WM CBF increasing at 40-minute post-exercise. ROI analyses showed decreased regional perfusion in the hippocampus and the insula at 10-minutes post-exercise. The final analysis on the resting perfusion images was done at a voxel-wise level and showed increased perfusion in the left somatosensory region at 40 minutes after exercise compared to pre-exercise. Although the fMRI group activations during a “go/no-go” task were highly robust, the only session effect that was detected was found in the left parietal operculum, with significantly reduced activation post-exercise.

Global CBF showed a statistically significant 10% decrease in GM at post-10 followed by a return to pre-exercise levels at post-40. These results are at odds with one other ASL acute exercise study [26], which may be explained by differences in end-tidal CO_2_ subsequent to exercise but is more likely due to the absolute ASL quantification adopted in the current study. Changes in blood pressure, which was lower 10 minutes post-exercise compared to before exercise or 40 minutes after exercise, may also influence global perfusion [29], [30]. During MRI, we monitored heart rate and blood pressure, but did not monitor breathing rate, O_2_ or CO_2_ levels, which is a limitation of this study. As discussed below, however, our findings highlighted that the brain’s response to exercise appears to depend on tissue type (i.e. GM vs WM) and brain region.

Interestingly, global WM CBF was increased at both perfusion time points after exercise compared to pre-exercise levels, which was a different response compared to global GM CBF. This discrepancy may reflect the following potential acute exercise effects. First, changes in CO2-related vascular tone can impact GM and WM differently [31]; others have reported cerebrovascular reactivity is altered due to chronic exercise [12], [13] and may be implicated in acute exercise [26]. Second, acute CBF increases in WM may contribute to an exercise-related improvement in WM integrity that has been reported [32]. Third, post-exercise regional allocation of CBF may reflect changes in functional connectivity since others report acute exercise impacts attention-related brain networks [33]. Finally, although our ASL protocol was conducive to measuring global WM CBF [22], future work that addresses WM perfusion using ASL or other modalities would corroborate our findings.

Although the ROI analysis showed a consistent trend of initial decrease in CBF 10 minutes after exercise followed by a CBF rebound increase at 40 minutes after exercise, only the hippocampus and insula ROIs showed significant differences between time points. The hippocampus has been reported in chronic exercise studies [5], [14] and the insula is reported in a CBF study of active and passive cycling [34] as well as volumetric changes associated with a sedentary lifestyle [35]. The hippocampal and insula regions are particularly sensitive to the effects of exercise, such as metabolic, ventilation, heart rate, blood pressure, or blood glucose changes [36], [37]. In the pioneering work by Williamson et al, perfusion-based single photon emission computed tomography (SPECT) was used along with MRI to localize an acute effect of exercise in the insula and leg sensorimotor regions [34]. With respect to autonomic effects, such as the elevated blood pressure 10 minutes after exercise, CBF in both the hippocampus and insula are reportedly associated with blood pressure [38]. Strategies that improve CBF quantification may help to increase the regional sensitivity to detect a session effect, since changes in arterial transit time for example are known to influence CBF [28].

Voxel-wise analysis identified increased CBF in the left lower limb somatosensory region at 40 minutes post exercise [39], but this region was found to border grey and white matter. The lower limb sensorimotor region was previously identified using both positron emission tomography [40] and single photon emission computed tomography studies during exercise [34]. In the current study it is not possible to say whether the increased CBF is due to a carry-over effect after cessation of exercise, an introspective motor imagery effect [41] or both.

BOLD fMRI data showed a regionally specific session effect that may be related to exercise. Specifically, the left parietal operculum had lower BOLD percent change activation after exercise compared to pre-exercise, which remained significant after controlling for resting perfusion. The findings in the parietal operculum may reflect either a reduction in the demand to coordinate the visuomotor response [42], a decrease in the need to attend to the stimuli [43], or vestibular or autonomic nervous system effects [44].

We did not observe an effect of session on the behaviour “go/no-go” data, which was unexpected given that the task is designed to test attention and executive function capcity, which others have found to be sensitive to exercise [1], [15], [17], [45]. It does, however, appear that the acute effect of exercise on cognitive processes may be influenced by multiple factors and the literature has examples that are both in line with [46] and are at odds with [17], [47] with the current study. Limitations to our results regarding the behavioural data include potential practice effects and change in inter-subject strategy across sessions.

## Conclusion

Our findings indicate that a single bout of aerobic exercise leads to acute changes in resting cerebral perfusion and activation associated with a “go/no-go” task. This study highlights that the acute effects of exercise appear to have a robust cerebrovascular effect that varied in time and brain region, as seen by ASL perfusion. However these effects did not dramatically alter the brain’s activation, as seen by the BOLD fMRI. This study helps to characterize the impact that exercise has on the brain, suggesting that exercise provides acute stimuli that may contribute to neuroplastic, autonomous effects.
